# Cold Water Fish Gelatin Methacryloyl Hydrogel for Tissue Engineering Application

**DOI:** 10.1371/journal.pone.0163902

**Published:** 2016-10-10

**Authors:** Hee Jeong Yoon, Su Ryon Shin, Jae Min Cha, Soo-Hong Lee, Jin-Hoi Kim, Jeong Tae Do, Hyuk Song, Hojae Bae

**Affiliations:** 1 College of Animal Bioscience and Technology, Department of Bioindustrial Technologies, Konkuk University, Hwayang-dong, Kwangjin-gu, Seoul, 05029, Korea; 2 Center for Biomedical Engineering, Department of Medicine, Brigham and Women’s Hospital, Harvard Medical School, Cambridge, Massachusetts, 02139, USA; 3 Medical Device Research Center, Research Institute for Future Medicine, Samsung Medical Center, Seoul, Republic of Korea; 4 Samsung Biomedical Research Institute, Samsung Advanced Institute of Technology, Samsung Electronics Co., Ltd., Seoul, Republic of Korea; 5 Department of Biomedical Science, CHA University, Bundang-gu, Gyeonggi-do, Republic of Korea; 6 College of Animal Bioscience and Technology, Department of Stem Cell and Regenerative Biology, Konkuk University, Hwayang-dong, Kwangjin-gu, Seoul, 05029, Korea; Brandeis University, UNITED STATES

## Abstract

Gelatin methacryloyl (GelMA) is a versatile biomaterial that has been used in various biomedical fields. Thus far, however, GelMA is mostly obtained from mammalian sources, which are associated with a risk of transmission of diseases, such as mad cow disease, as well as certain religious restrictions. In this study, we synthesized GelMA using fish-derived gelatin by a conventional GelMA synthesis method, and evaluated its physical properties and cell responses. The lower melting point of fish gelatin compared to porcine gelatin allowed larger-scale synthesis of GelMA and enabled hydrogel fabrication at room temperature. The properties (mechanical strength, water swelling degree and degradation rate) of fish GelMA differed from those of porcine GelMA, and could be tuned to suit diverse applications. Cells adhered, proliferated, and formed networks with surrounding cells on fish GelMA, and maintained high initial cell viability. These data suggest that fish GelMA could be utilized in a variety of biomedical fields as a substitute for mammalian-derived materials.

## Introduction

Hydrogels are composed of hydrophilic polymer networks crosslinked by chemical reactions such as covalent bonding, ionic bonding, hydrogen bonding, hydrophobic interactions, and crystallizing segments as well as protein interactions, etc. Diverse polymerization techniques facilitate hydrogel synthesis and control of their physical properties (such as degradation, stiffness, porosity, and swelling), to which encapsulated cells respond in terms of viability, proliferation, differentiation, and spreading. To date, a variety of naturally sourced and synthetic polymer-based materials have been employed as hydrogels for biomedical applications, including regenerative medicine, drug delivery, and tissue engineering [1–4].

For biomedical applications, naturally sourced polymers have advantages over synthetic polymers in terms of their low immunoresponse, high biocompatibility, and available bioactive motifs in the polymer [2, 5, 6]. Gelatins are produced by partial hydrolysis of native collagen, a major component of the extracellular matrix (ECM) in most animal tissues, and have been widely used in the food, pharmaceutical, cosmetic, and photography industries [7, 8]. They contain abundant arginine-glycine-aspartic acid (RGD) sequences, which promote cell adhesion, and target sequences of matrix metalloproteinases (MMP), which facilitate ECM remodeling [2, 9, 10]. As denatured collagens, gelatins have excellent solubility, low antigenicity and a low gelling point, but have a low mechanical modulus and undergo rapid degradation [2, 5, 6, 8, 10, 11].

To compensate for such disadvantages, chemically modified gelatin, gelatin methacryloyl (GelMA), can form photopolymerized hydrogels through UV irradiation in the presence of a photoinitiator [2]. Also, GelMA enables production of structures with various patterns or morphologies using micromolding or photopatterning techniques [9, 12]. Furthermore, 3D structures can be fabricated by stereolithography [13–15] for studies of cell-biomaterial interactions and control of cell behavior [16–19].

The physiological activities, mechanical properties and morphological changes of cells cultured on or within GelMA hydrogels have been investigated. For example, porcine gelatin based-GelMA and GelMA-microgels incorporating carbon nanotubes, gold nanoparticles and graphene oxides [4, 9, 20–22]. Although GelMA hydrogels or hybrids with other functional materials modulate cellular responses, clinical application of these hydrogels from mammalian sources must take into consideration the potential for zoonosis (e.g., Bovine Spongiform Encephalopathy) [8, 10, 23].

Given the background, fish gelatin has emerged as a useful biomaterial that could substitute for mammalian gelatin. In recent years, research related to fish gelatin extraction and its application in biomedical engineering has increased due to its advantages over mammalian gelatin. First, economical production due to use of discarded byproducts of routine fish processing, unlike mammalian gelatin, the cost of which is influenced by that of raw materials [7]; and second, fewer personal or religious restrictions (e.g., vegetarianism, Judaism, Islam and Hinduism), who may be reluctant to use mammalian-origin biomaterials [7, 24–27].

In this study, we synthesized fish gelatin-based GelMA hydrogel using a conventional UV polymerization method after introducing a methacrylamide group to fish gelatin. The fish GelMA was compared with porcine GelMA in terms of physical properties (elastic modulus, degradation and water swelling) and cell behavior (viability, proliferation and spreading). The results suggest the feasibility of use of fish GelMA as a substitute for mammalian GelMA in drug delivery, regenerative medicine and tissue engineering.

## Materials and Methods

### Materials

Gelatin from porcine skin (Type A, 300 bloom), gelatin from cold-water fish skin, methacrylic anhydride (MA), 2,4,6-trinitrobenzene sulfonic acid solution (TNBS) and 3-(trimethoxysilyl)propyl methacrylate (TMSPMA) were purchased from Sigma-Aldrich (Wisconsin, USA). Microscope slides and cover glasses were purchased from Marienfeld-Superior (Lauda-Königshofen, Germany). Culturewell^™^ Chambered Coverslips were purchased from Grace Bio-Labs (Oregon, USA). The UV light source (Omnicure S2000) was purchased from EXFO Photonic Solutions Inc. (Ontario, Canada). Spacer thickness was measured using electronic dial calipers (Mitutoyo Co, Tokyo, Japan).

### Synthesis of gelatin methacryloyl (GelMA)

**Fig 1:**
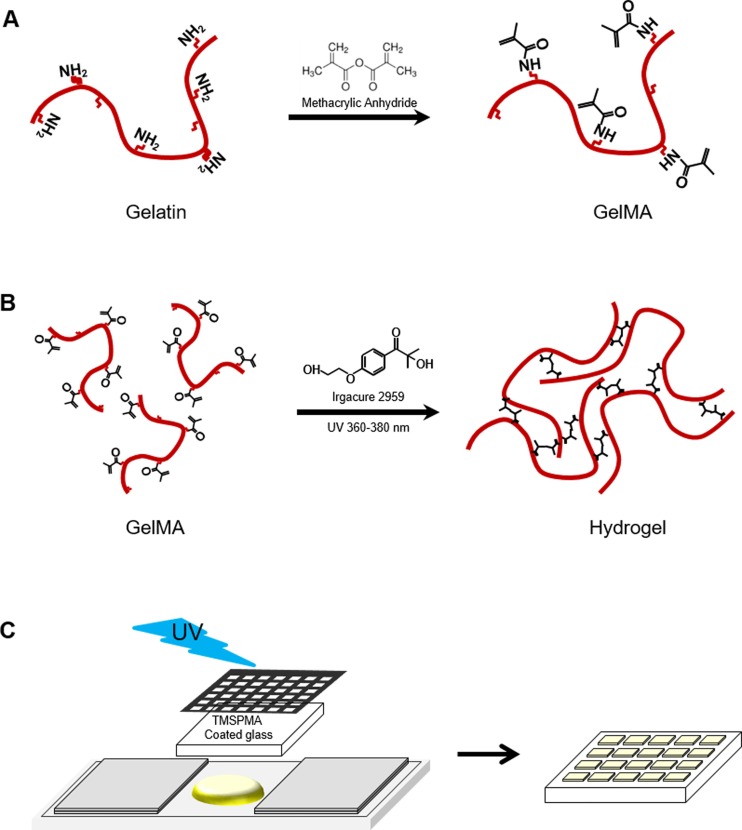
Synthesis of fish gelatin methacryloyl (GelMA) and fabrication of photocrosslinked GelMA hydrogel. (A) Gelatin was reacted with methacrylic anhydride (MA) to introduce a methacryloyl substitution group on the reactive amine and hydroxyl groups of the amino acid residues. (B) GelMA photocrosslinking to form A hydrogel matrix under UV irradiation. The free radicals generated by the photoinitiator initiated chain polymerization with methacryloyl substitution. (C) Schematic of formation of patterned hydrogels using photolithography.

Fish and porcine GelMA was synthesized as described previously (Fig 1A) [2, 9]. Gelatin was mixed at 10% (w/v) in phosphate-buffered saline (PBS; Welgene, Korea) (50°C) and stirred until fully dissolved. Methacrylic anhydride was added until the target volume (0.25, 1.25, and 20% (v/v) of MA) was reached at a rate of 0.5 ml/min to the gelatin solution with stirring at 50°C and allowed to react for 2 h. The reactive amine and hydroxyl groups of the amino acid residues were modified by changing the amount of MA present in the initial reaction mixture. After dilution (5×) with warm (40°C) PBS to stop the reaction, the mixture was dialyzed against distilled water using 12–14 kDa cutoff dialysis tubing for 1 week at 40°C to remove low-molecular-weight impurities (including unreacted MA and methacrylic acid byproducts, etc.), which are potentially cytotoxic. The solution was lyophilized for 7 days to generate a white porous foam and stored at 4°C until further use.

### Evaluation of degree of methacrylation

The degree of methacrylation was measured using a method developed by Habeeb with TNBS as described previously [28, 29]. Briefly, freeze-dried GelMA was dissolved at 200 μg/ml (w/v) in 0.1 M sodium bicarbonate (pH 8.5). Then, 0.25 ml of 0.01% (w/v) TNBS solution was added to 0.5 ml of each sample solution and samples were incubated at 37°C for 2 h. To stop and stabilize the reaction, 0.25 ml of 10% sodium dodecyl sulfate (SDS) and 0.125 ml of 1 N hydrogen chloride (HCl) were added to each sample. Optical density (OD) was determined using a UV/vis spectrophotometer (OPTIZEN POP, Mecasys, Korea) at 335 nm. The extent of substitution was calculated by comparing the amount of remaining free amino groups in gelatin and GelMA.

### Preparation of prepolymer solution

Lyophilized GelMA macromer was dissolved in PBS containing 0.5% (w/v) 2-hydroxy-1-(4-(hydroxyethoxy)phenyl)-2-methyl-1-propanone (Irgacure 2959, CIBA Chemicals, Tarrytown, NY) as a photoinitiatior at 80°C, and subsequently used for fabrication of hydrogels.

### Mechanical testing

Prepolymer (200 μl) was pipetted on to PDMS mold (10 mm (diameter) × 2.5 mm (thickness)) and exposed to 7.3 mW/cm^2^ UV light (360–380 nm) for 100 s (Fig 1B). Samples were incubated at room temperature in PBS for 24 h. The hydrogel discs were tested using a CT3 Texture Analyzer with a 4500 g load cell (Brookfield Engineering Laboratory, Stoughton, MA) in compression mode. A probe of 12.7 mm diameter was used to compress the samples at 0.01 mm/s. A stress-strain curve was obtained and compressive modulus was determined as the slope of the linear region corresponding to 5–15% strain. The number of samples was three per group.

### Swelling test

Prepolymer (200 μl) was pipetted between two slide glass separated by a 1 mm spacer and exposed to 7.3 mW/cm^2^ UV light (360–380 nm) for 60 s. Immediately after hydrogel formation, each sample was placed in PBS at 37°C for 24 h. Excess PBS was removed and the weight of the swollen hydrogel was recorded. Samples were then lyophilized and the dried polymer weighed. The mass-swelling ratio was calculated as the ratio of swollen hydrogel mass to the mass of dry polymer. The number of samples was five per group.

### Hydrogel degradation

Prepolymer (30 μl) was pipetted on to Culturewell™ chambered coverslips (6 mm (diameter) × 1 mm (thickness)) and exposed to 7.3 mW/cm^2^ UV light (360–380 nm) for 60 s. Hydrogels were placed in 1.5 ml tubes containing 1 ml of PBS with 2 U/ml collagenase type II (Worthington Biochemical Corp., Freehold, NJ). Gels were incubated with collagenase type II at 37°C for 1.5, 3, 6, 9, 12, 18 and 24 h. At each time point, the collagenase solution was removed without disturbing the undigested hydrogel. The remaining hydrogel was washed with distilled water, all liquid was removed and gels were lyophilized. Subsequently, Dried hydrogels were subjected to morphological analysis by scanning electron microscopy (SEM). The percent degradation was calculated by the dried weight after digestion divided by the weight of untreated hydrogels. The sample size was three per group. Pore size frequency (%) was obtained from 5 SEM images per condition and calculated using ImageJ 1.50a software (National Institutes of Health, Bethesda, Maryland).

### SEM Imaging

To confirm the morphology of degraded hydrogels, SEM images of lyophilized hydrogels after degradation were taken. The samples were cut to expose their cross-sections and coated with platinum using a sputter coater (Hitachi E-1030, Japan). Sample cross-sections were imaged using an SEM (Hitachi Model S-4300, Japan).

### Cell culture

NIH3T3 fibroblasts purchased from Korean Cell Line Bank (KCLB) were cultured in high-glucose Dulbecco’s Modified Eagle’s Medium (DMEM; Welgene, Daegu, Korea) supplemented with 10% FBS and 1% penicillin-streptomycin in a 5% CO_2_ atmosphere at 37°C, and passaged twice per week.

### Cell characterization

For cell adhesion studies, cylinder hydrogel (6 mm (diameter) × 1 mm (thickness)) were prepared in a similar manner as for degradation testing. We also fabricated micropatterned hydrogels (800 × 800 μm) using a photomask process [30] following exposure to 7.3 mW/cm^2^ UV light (360–380 nm) for 40 s (Fig 1C). These micropatterned hydrogel were utilized for assessing acute 2D cytotoxicity within the normalized area. All hydrogels were produced on TMSPMA-coated glass slides. NIH3T3 fibroblasts (2×10^5^ cells/ml) were trypsinized and resuspended in medium. Cell suspension was added to hydrogels, and incubated for 2 h. Medium was changed every 24 h for 5 days. To evaluate cell viability, cells were stained after 24 h using a calcein-AM/ethidium homodimer Live/Dead assay kit (Invitrogen, Carlsbad, CA) according to the manufacturer’s instructions. The fluorescence microscope (Nikon, ECLIPSE Ts2, Japan) was used to obtain the live and dead image and the number of live and dead cells from 3 randomly selected microgel units of three patterns of each GelMA group. The number of live and dead cells were counted using ImageJ 1.50a software. After 1 and 3 days, cells were stained with FITC-labeled phalloidin (Sigma) at 1:100 dilutions in blocking buffer for 100 min and DAPI (Sigma) at 1:1000 dilution in DPBS for 5 min to visualize F-actin filaments and nuclei, respectively. The stained samples were visualized using a fluorescence microscope. To evaluate cell proliferation on GelMA hydrogels, MTS assays (Celltiter 96 Aqueous One Solution, Promega, USA) were performed according to the manufacturer's instructions using three samples per group.

For 3D cell encapsulation studies, NIH3T3 were resuspended in media at a concentration of 4×10^6^ cells/ml and mixed into prepolymer containing 10% (w/v) GelMA and 1% (w/v) photoinitiator. The mixture containing 2×10^6^ cells/ml of cells, 0.5% of photoinitiator and 5% GelMA was pipetted between two microscope slides separated by a 150 μm spacer and exposed to 7.3 mW/cm^2^ UV light (360–380 nm) for 15 s on TMSPMA treated glass. The glass slides containing hydrogels were washed with DPBS and incubated for 5 days in NIH3T3 medium under standard culture conditions, with the media being changed every 24 h. After the cell encapsulation, a calcein-AM/ethidium homodimer Live/Dead assay was used to quantify cell viability within the hydrogels (3 h and 24 h). The images of live and dead cells were obtained from 3 randomly selected spots of three cylindrical hydrogels of each GelMA group. After 3 days of culture, encapsulated cells in hydrogels were stained with FITC-labeled phalloidin (green) at 1:100 dilutions in blocking buffer for 100 min and DAPI (blue) at 1:1000 dilutions in DPBS for 5 min for cytoskeleton staining. For cell proliferation measurement, encapsulated cell was subject to MTS assay according to the manufacturer’ instructions using three samples per group.

### Statistical Analysis

To evaluate statistical significance, a one- or two-way ANOVA followed by Bonferroni's post-hoc test was performed. Data is means ± standard deviation (SD) and means were compared using unpaired Student’s t-tests. A *p*-value < 0.05 was considered to indicate statistical significance. All analyses were performed using GraphPad Prism 5.01 (GraphPad Software, La Jolla, CA).

## Results and Discussion

### Degree of methacrylation

**Fig 2:**
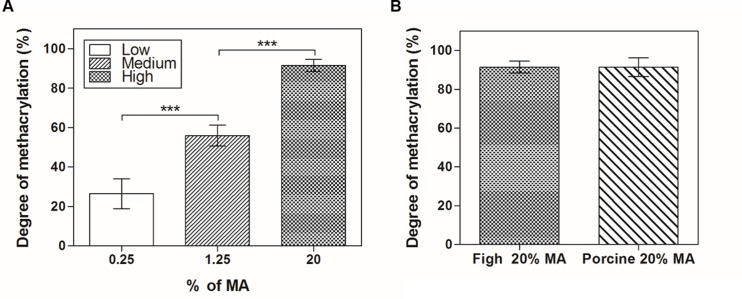
Degree of methacrylation as determined by TNBS assay. (A) Various volume percentages of methacrylic anhydride (0.25%, 1.25% and 20%) were analyzed to investigate the degree of methacrylation of the synthesized fish GelMA. (B) Comparison of a high degree of methacrylation (20% MA) according to the origin of gelatin (fish GelMA vs. porcine GelMA); there was no significant difference. The percentage of incorporated substitution was calculated by comparing the amount of remaining amino groups (-NH_2_) in GelMA to that in pristine gelatin. Error bars represent standard deviations (SDs) of measurements performed on six samples.

GelMA with various degrees of methacrylation were synthesized using different concentrations of MA in PBS at 50°C. Since MA bonds mainly to reactive amine groups on the polypeptide backbone [9], the degree of methacrylation of GelMA was quantified by TNBS assay; the results confirmed the extent of substitution of free amine groups in gelatin and GelMA chains. We obtained fish GelMA with low (26.4 ± 7.5%), medium (55.9 ± 5.3%) and high (91.4 ± 3.1%) degrees of methacrylation by adding 0.25%, 1.25% and 20% (v/v) MA (Fig 2A). Therefore, fish GelMA with 20–90% methacrylation were successfully produced. A control sample comprising porcine GelMA with a high degree of methacrylation (91.3 ± 4.8%) did not show only significant difference when compared with fish GelMA in degree of methacrylation (Fig 2B).

### Mechanical properties

**Fig 3:**
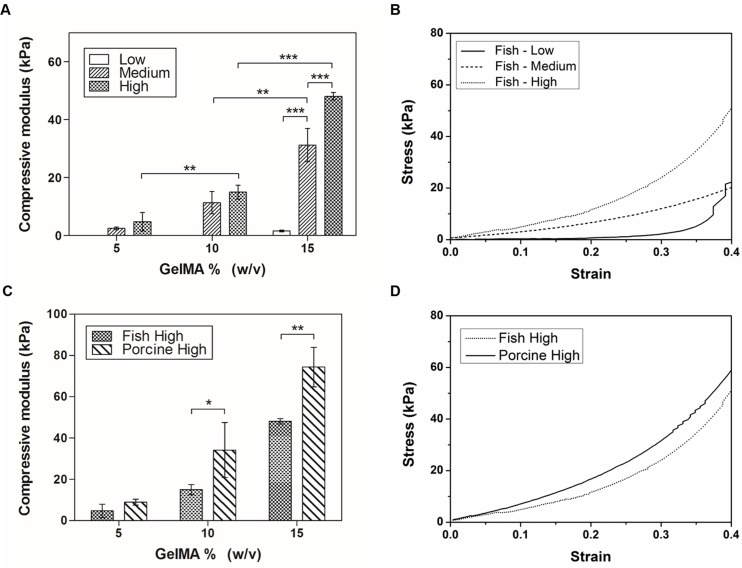
Mechanical properties of fish GelMA hydrogels with various degrees of methacrylation and gel percentages. (A) Compressive modulus for 5%, 10% and 15% (w/v) fish GelMA at low, medium and high degree of methacrylation, with the exception of low degree, 5% and 10% (w/v) GelMA which formed gels too weak to be handled for testing. (B) Representative stress-strain curve of a 15% (w/v) GelMA according to degree of methacrylation. (C) Comparison of fish and porcine GelMA hydrogels with a high degree of methacrylation. (D) Representative curve of 15% (w/v) GelMA for fish and porcine GelMA comparison. Conditions of 10% and 15% (w/v) GelMA were significantly different (*p<0.05, **p<0.01, ***p<0.001). Error bars represent SDs of measurements performed on 3 samples.

The mechanical properties of the ECM influence cell behavior, function and differentiation [31]. To determine the effect of polymer concentration and methacrylation degree on the mechanical properties of fish GelMA hydrogels, unconfined compression tests were performed using swollen hydrogels. The compressive modulus of fish GelMA hydrogels with a high degree of methacrylation was significantly higher than that of fish GelMA hydrogels with low and medium degrees of methacrylation (Fig 3A). In addition, the compressive modulus of fish GelMA hydrogels increased with increasing polymer concentration. The 5% and 10% (w/v) GelMA with a low degree of methacrylation could not be tested because they were too weak to be handled. The stress-strain curve for the 15% (w/v) GelMA hydrogel shows that increasing the degree of methacrylation increased the stiffness at all strain levels for all three gel concentrations (Fig 3B). We investigated the compressive moduli of fish and porcine GelMA at 5%, 10% and 15% with a high degree of methacrylation (Fig 3C and 3D). The compressive moduli of fish and porcine GelMA hydrogels significantly differed at 10% and 15% GelMA concentration (Fig 3C). The low strength of fish GelMA hydrogels was due to their different amino acid composition and molecular weight distribution [26, 32–35]. Gelatin from cold-water fish skin and mammalian gelatin contain the same quantity of lysine, which is a major methacrylation site. However, cold-water fish gelatin has fewer hydrophobic amino acids (alanine, valine, leucine, isoleucine, proline, phenylalanine, and methionine) and imino acids (proline and hydroxyproline) than mammalian gelatin [36, 37]. Therefore, even at a similar degree of methacrylation and photopolymerization conditions, gels produced using fish GelMA may have insufficient hydrophobic interactions, and could be easily deformed even by a small force due to having relatively lower number of imino acids which provide structural stability. Therefore, the expected strength of hydrogels based on fish gelatin is lower.

### Swelling characteristic

**Fig 4:**
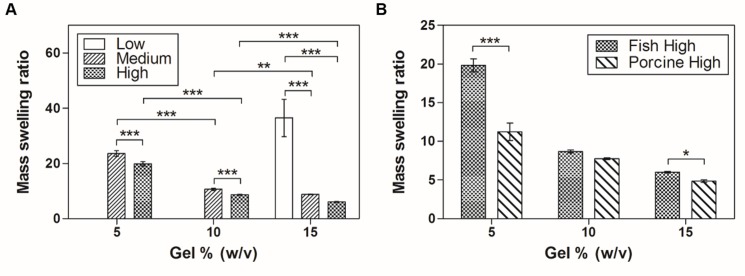
Equilibrium swelling properties of GelMA hydrogels. (A) The mass swelling ratios of fish GelMA hydrogels containing 5%, 10% and 15% (w/v) GelMA and low, medium and high degrees of methacrylation were significantly different (*p<0.05, **p<0.01, ***p<0.001). GelMA (5% and 10% (w/v)) with low methacrylation produced hydrogels which were too weak to be handled and therefore were not tested. (B) Comparison of fish and porcine GelMA hydrogels with a high degree of methacrylation. Error bars represent SDs of measurements of five samples.

The swelling characteristic of hydrogels is important as it influences solute diffusion and mechanical properties [9]. The swelling behavior of hydrogels depends on their structural properties, such as their interaction with the solvent, cross-linking density and hydrophilicity [38, 39]. To investigate the swelling behavior, we fabricated fish GelMA hydrogels using diverse conditions (low, medium and high degree of methacrylation and 5%, 10% and 15% (w/v) GelMA). All gels were fully swelled in DPBS at 37°C for 24 h. Then, the mass-swelling ratio of the swollen sample to the dry mass of polymer was calculated. However, we could not obtain the swelling ratio of the 5% and 10% (w/v) fish GelMA with a low degree of methacrylation due the structural weakness of the gels produced. The mass-swelling ratio increased with decreasing degree of methacrylation and/or GelMA concentration, as has been reported previously [9, 40] (Fig 4A). To compare the swelling ratios of fish and porcine GelMA with a high degree of methacrylation, porcine GelMA hydrogels were fabricated using the same method as for fish GelMA hydrogels (Fig 4B). The mass-swelling ratios of porcine GelMA gels were similar to those of fish GelMA hydrogels expect for 5% (w/v) GelMA concentration. The values of 5% (w/v) fish and porcine GelMA were 19.8 ± 0.8 and 11.2 ± 1.1, respectively, a twofold difference. Fish GelMA showed a higher swelling ratio than porcine GelMA, with the exception of 10% (w/v) gel. The swelling properties of the fish GelMA hydrogel are dependent mainly on the pore size, the crosslinking density of the polymer network and the interaction between the polymer and solvent [38–41]. In this respect, the swelling degree of hydrogel is inversely proportional to the gel concentration because the polymer network density increases with increasing gel concentration [40]. This assumption is supported by the mechanical testing result that fish GelMA formed a softer hydrogel than porcine GelMA (Fig 3D). In addition, the fish gelatin has higher number of hydrophilic amino acid relative to mammalian gelatin resulting in increased swelling ratio of fish GelMA hydrogel compared with porcine GelMA gels [36, 37].

### Degradation profiles

**Fig 5:**
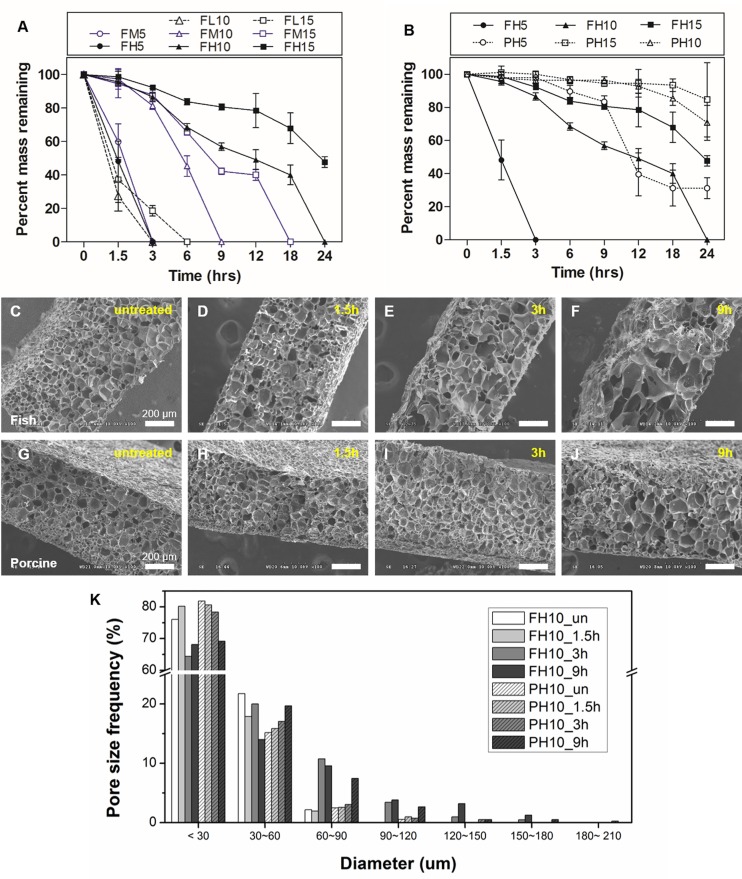
Degradation characteristics of fish GelMA hydrogels. (A) Degradation profiles of fish GelMA hydrogels with various degrees of methacrylation (low, medium and high) and GelMA concentrations (5%, 10% and 15%) upon exposure to collagenase type II. (B) Comparison of fish and porcine GelMA hydrogels with high degree of methacrylation and 10% gel concentration. Error bars represent SDs of measurements performed on three samples. Representative cross-sectional SEM images of fish GelMA (C~F) and porcine GelMA (G~J) hydrogels reveal different gel morphologies after degradation with collagenase type II. (K) Pore size distribution of GelMA hydrogels (Pore size frequency obtained from 5 SEM images per condition).

The degradation properties of hydrogels play an important role in creation of a cellular microenvironment appropriate for 3D tissue engineering. In particular, GelMA retains target sequences of matrix metalloproteinases (MMPs) [2]. Therefore, cells encapsulated in GelMA hydrogels can degrade and remodel the surrounding hydrogel by replacing it with cell-secreted ECM [2, 28, 42–44]. To confirm and compare the degradation profile of fish and porcine GelMA hydrogels, enzyme-mediated degradation tests were performed using various degrees of methacrylation and gel concentrations. All samples were immersed in 2 U/ml of collagenase type II solution for 0, 1.5, 3, 6, 9, 12, 18 and 24 h. The degradation rate of fish and porcine GelMA hydrogels decreased as the gel concentration increased (Fig 5A and 5B). The fish GelMA hydrogel completely degraded within 24 h under all conditions, with the exception of 15% (w/v) fish GelMA with a high degree of methacrylation with 47.7 ± 3.1% of hydrogel remaining (Fig 5A). Regarding fish and porcine GelMA (10% (w/v)), 95.6 ± 2.2% and 56.8 ± 2.2% of the fish GelMA remained after 1.5 and 9 h, respectively, compared to 97.5 ± 2.1% and 96.3 ± 2.1%, respectively, for porcine GelMA (Fig 5B). Scanning electron microscopy was performed to confirm the difference in degradation of 10% GelMA hydrogels (Fig 5C–5J). The cross-sectional SEM image revealed different morphologies between the two samples after degradation. After 9 h, porcine GelMA maintained an ordered and porous microstructure (Fig 5J) compared to the collapsed, disordered microstructure of degraded fish GelMA (Fig 5F). Furthermore, untreated fish and porcine GelMA hydrogels had similar average pore diameters (22.4 ± 2.2 μm and 17.5 ± 2.5 μm, respectively), but after 9 h, fish GelMA hydrogels resulted in a larger pore diameter compared to porcine hydrogels (36.2 ± 9.4 μm and 26.5 ± 11.1 μm, respectively). The high degradation rates of fish GelMA hydrogel may be due to the unique amino acid composition of fish gelatin. Fish gelatin contains fewer content of imino acid that provide structural stability compared to porcine gelatin [36, 37], resulting in lower gel strength and higher water swelling properties (Figs 3 and 4). In this context, fish gelatin has a lower structural stability enabling rapid infiltration of the enzyme into the hydrogel, causing faster degradation rates. Thus, cells encapsulated within the 10% and 15% porcine GelMA hydrogels could not readily spread and proliferate in, and degrade the hydrogel [9], because such gels exhibited a smaller and dense pore network. However, fish GelMA hydrogels have a lower mechanical modulus with a lower structural stability than porcine GelMA gels at the same gel concentration. Therefore, encapsulated cells in fish GelMA hydrogels might exhibit superior spreading, migration, and proliferation than those in porcine GelMA hydrogels.

### Cell adhesion to 2D GelMA surfaces

**Fig 6:**
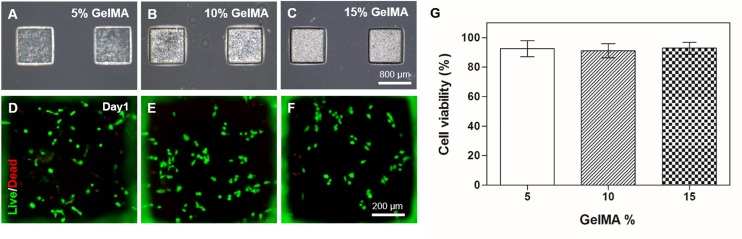
Fabrication of micropatterned fish GelMA hydrogel and viability of cells on micropatterned gel surfaces. NIH3T3 cells readily adhered to fish GelMA surfaces irrespective of macromer concentration. (A-C) Pattern fidelity of fish GelMA using 5%, 10% and 15% macromer (scale bar = 800 μm). (D-F) LIVE/DEAD assay at 24 h after adhesion (scale bar = 200 μm). (G) Quantification of cell viability demonstrated high cell survival under all conditions and there was no significant difference between GelMA conditions. Error bars represent SDs of averages obtained from five images per condition.

**Fig 7:**
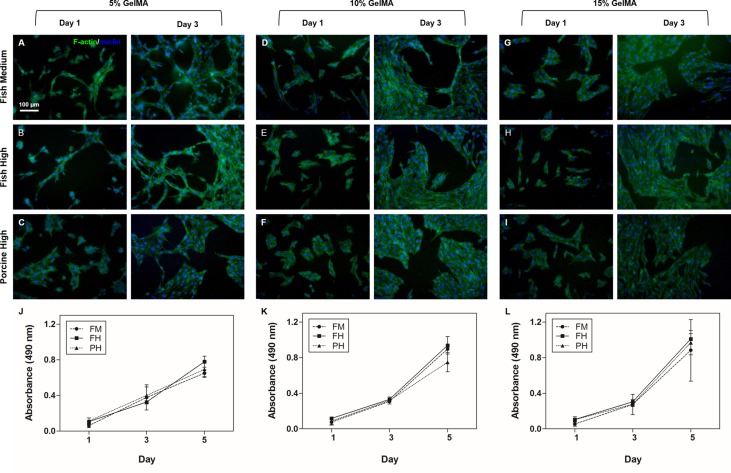
Cell adhesion and proliferation on fish GelMA surfaces. NIH3T3 cells on 5%, 10% and 15% (w/v) GelMA with a medium and high degree of methacrylation adhered and proliferated (day 5). (A-I) Representative images of NIH3T3 cells on fish GelMA surfaces stained with phalloidin (green)/DAPI (blue) on day 1 and day 3 of culture (scale bar = 100 μm). (J-L) MTS assay of cells on GelMA hydrogels after 1, 3 and 5 days. Error bars represent SDs of averages of three samples per condition. There were no significant differences between fish and porcine GelMA.

Biomaterials have been utilized in various applications, such as artificial vessels and bones, which are directly inserted into the body to repair or replace damaged tissue. Therefore, assessment of cell behavior (viability, adhesion and proliferation) on biomaterials is crucial [45, 46]. Fish GelMA hydrogel with a high degree of methacrylation were selected due to its ease of handling and microstructure forming ability (Figs 6 and 7). In Fig 6A–6C, fish GelMA at varying concentrations could be successfully micropatterned with high pattern fidelity. To evaluate cellular toxicity on the surface of the micropatterned fish GelMA hydrogels, NIH3T3 fibroblasts were seeded. NIH3T3 cells readily adhered to the surface of micropatterned fish GelMA hydrogels (800 × 800 μm) at all concentrations (Fig 6D–6F) and showed a viability of >90% at 24 h after attachment (Fig 6G). In addition, cells also readily adhered to the larger surface of fish GelMA hydrogel (diameter = 6 mm) of various concentrations (Fig 7). Seeded cells were elongated and formed interconnected cellular networks on fish GelMA hydrogel under all conditions on day 3 (Fig 7A, 7B, 7D, 7E, 7G and 7H). After 5 days of culture, fish GelMA hydrogels under all conditions were completely covered by cells (data not shown). In GelMA hydrogels with low mechanical moduli, notably 5% (w/v) gel concentration, cells separately elongated (Fig 7A–7C), while cells on hydrogels with high mechanical moduli were polygonal and interconnected with surrounding cells (Fig 7G–7I). The difference of cellular morphology between Figs 6 and 7 was caused by distinct stiffness properties of the respective hydrogel substrates (Fig 6D–6F and Fig 7B, 7E and 7H). To micropattern fish GelMA hydrogel, the UV curing time was adjusted to be shorter (40 s) than that of cylindrical fish GelMA hydrogel (60 s). The shorter curing time provided minimum cross-linking density required for the formation of hydrogel, thereby lowering the gel stiffness [21, 47] followed by different morphology of cells attached on the respective surfaces [48, 49]. Nonetheless, the difference of mechanical properties did not appear to affect the results of cytotoxicity testing of our material in this case. By day 5 of culture, cell proliferation increased fivefold compared to day 1 under all conditions, and there was no significant difference between samples at the same GelMA concentration (Fig 7J–7L). Natural proteins that contain RGD sequences and MMP-sensitive sequences are attractive materials for cell-reactive scaffolds [50, 51]. Use of natural-sourced protein, such as gelatin, would eliminate the effort required to incorporate cell-sensitive motifs in the engineered scaffold. Since fish GelMA hydrogels also possess cell binding sites (RGD sequence), cells readily bind to them (Figs 6 and 7). Furthermore, cells readily proliferated, elongated and formed interconnected networks with surrounding cells on the fish GelMA hydrogel (Fig 7). These data suggest that our fish GelMA hydrogel can be used as a highly biocompatible material in various biomedical fields.

### 3D cell encapsulation in GelMA

**Fig 8:**
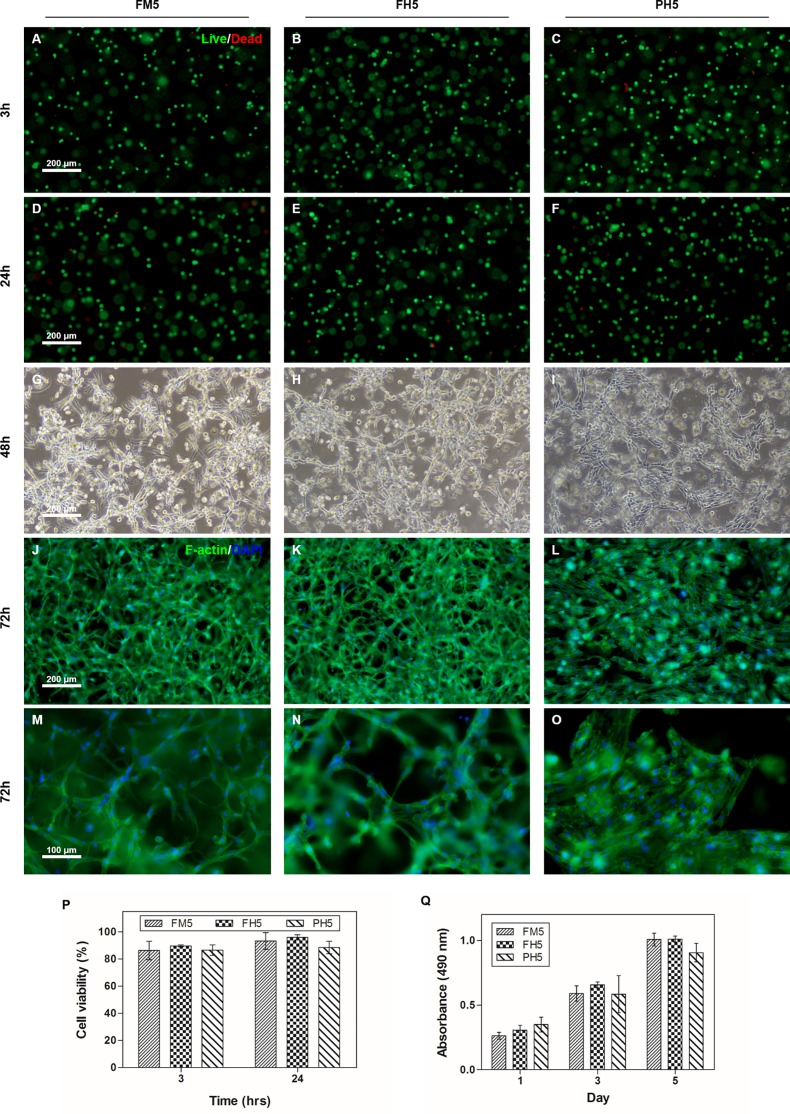
Behavior of cells encapsulated in fish GelMA hydrogels. (A-F) NIH3T3 cells embedded in medium and high degree of methacrylation fish GelMA containing 5% (w/v) GelMA were stained using calcein-AM/ethidium homodimer at 3 h and 24 h after encapsulation to evaluate the cell viability compared to high degree of methacrylation porcine GelMA. (G-I) After 2 days of culture, cells proliferated and elongated in fish and porcine GelMA hydrogel. (J-L) Representative images of the cells stained with phalloidin for the actin filaments (green) and nuclei counterstained with DAPI (blue) at 72 h. (P) Viability of the encapsulated cells. (Q) Quantification of cell proliferation in GelMA hydrogel until 5 days. Cell proliferation rate was not significantly different at all condition.

To examine the feasibility to employ our fish GelMA hydrogel to tissue engineering applications, NIH3T3 cells were encapsulated in the fish GelMA hydrogels (h = 150 μm). Hydrogels with 5% (w/v) gel were selected based on our previous data since cells embedded in 10% and 15% (w/v) GelMA hydrogels showed negligible activities in cell migration or degrading the hydrogels [9]. Encapsulated cells were cultured for 5 days and cell viability was assessed using the LIVE/DEAD assay kit (Fig 8A–8F). In results, cells in both fish and porcine GelMA hydrogels were highly viable during the culture period, maintaining high levels of viability at approximately 90%. (Fig 8P). After 2 days of culture, encapsulated cells elongated and formed interconnected networks regardless of the types of GelMA (Fig 8G–8I). The phalloidin staining results further confirmed the elongated and spread cell morphologies demonstrated in the microscopic observation data (Fig 8J–8O). However, there was a difference in cellular morphologies observed between fish and porcine GelMA hydrogels. As aforementioned, hydrogel stiffness can affect cellular morphologies such that cells encapsulated in the fish GelMA tended to be more elongated than those in the porcine GelMA due to its lower stiffness [4]. Meanwhile, routine cell proliferation was also observed in both fish and porcine GelMA hydrogel without significant differences as evaluated by the MTS assay during the culture period (Fig 8Q). Recently, various cell culture techniques in 3D scaffolds have been used because 2D cell cultures (monolayer form) cannot be used to investigate the behavior of native cells. The existing porcine GelMA has received much attention because it meets the requirements for biomaterials, such as biofunctionality and mechanical variability [2, 51]. In this regard, fish GelMA could also be used for 3D culture and show higher initial cell survival (Fig 8). These data suggest that fish GelMA could be useful as scaffolds for 3D cell culture.

## Conclusions

In this study, the characteristics of fish GelMA derived from cold-water fish gelatin and its potential for various applications were demonstrated. In particular, the physical properties of fish GelMA were tunable depending on the degree of methacrylation and the gel concentration. The compressive moduli of fish GelMA were relatively lower than those of porcine GelMA under identical conditions. However, the degradation rate was higher than that of porcine GelMA hydrogel. Since the melting and gelling points of fish GelMA were lower than those of porcine GelMA, fish GelMA exhibited easier handling even at high gel concentrations, and could readily incorporate heat-sensitive molecules. For instance, fish GelMA can be solubilized with ease at room temperature, while porcine GelMA requires heat and readily solidifies at RT at higher gel concentrations (> ~15%). Even at high gel concentrations, fish GelMA solution did not solidify, but remained a viscous fluid that underwent slow coagulation at room temperature. This capability renders fish GelMA suitable for diverse applications, such as medical adhesive or delivery of molecules that denature at high temperature (e.g., vitamins). Furthermore, fish GelMA may have potential as a bio-ink for 3D printing application, because it readily melts at the temperature typically used for cell culture (~37°C). Fish GelMA also showed high fidelity for microscale-patterning applications. Cells cultured in fish GelMA hydrogel showed high viability and proliferation and formed networks with surrounding cells. For 3D encapsulation, cells in fish GelMA hydrogel also retained high viability until 24 h after encapsulation and proliferation rate of encapsulated cells in fish GelMA for 5 days was comparable with cells encapsulated in porcine GelMA. These data suggest that gelatin from cold-water fish may be used to develop engineered biomaterials for drug delivery, regenerative medicine and tissue engineering.

## Supporting Information

S1 Fig The raw data for TNBSA test (Fig 2). (A) Degree of methacrylation of fish GelMA (Fig 2A). (B) Comparison of a high degree of methacrylation (20% MA) according to the origin of gelatin (fish GelMA vs. porcine GelMA) (Fig 2B).
(XLSX)

S2 Fig The raw data for mechanical test (Fig 3). (A) Compressive modulus for fish GelMA hydrogels (Fig 3A). (B) Representative stress-strain curve of fish GelMA hydrogels (Fig 3B). (C) Comparison of fish and porcine GelMA hydrogels (Fig 3C). (D) Representative curve of 15% (w/v) GelMA for fish and porcine GelMA comparison (Fig 3D).
(XLSX)

S3 Fig The raw data for swelling test (Fig 4). (A) The mass swelling ratios of fish GelMA hydrogels (Fig 4A). (B) Comparison of fish and porcine GelMA hydrogels (Fig 4B).
(XLSX)

S4 Fig The raw data for hydrogel degradation test (Fig 5). (A) Degradation profiles of fish GelMA hydrogels (Fig 5A). (B) Comparison of fish and porcine GelMA hydrogel (Fig 5B). (C) Pore size of GelMA hydrogels (Fig 5K).
(XLSX)

S5 Fig The raw data for cell viability test of 2D cell culture (Fig 6). (XLSX)

S6 Fig The raw data for cell proliferation test of 2D cell culture (Fig 7). (A-C) MTS assay of cells on GelMA hydrogel (Figs J-K).
(XLSX)

S7 Fig The raw data for 3D cell culture test (Fig 8). (A) Cell viability of the encapsulated cells (Fig 8P). (B) Quantification of cell proliferation in GelMA (Fig 8Q).
(XLSX)

## References

[1] Peppas NA, Hilt JZ, Khademhosseini A, Langer R. Hydrogels in Biology and Medicine: From Molecular Principles to Bionanotechnology. Advanced Materials. 2006;18(11):1345–60. 10.1002/adma.200501612

[2] Yue K, Trujillo-de Santiago G, Alvarez MM, Tamayol A, Annabi N, Khademhosseini A. Synthesis, properties, and biomedical applications of gelatin methacryloyl (GelMA) hydrogels. Biomaterials. 2015;73:254–71. 10.1016/j.biomaterials.2015.08.045 26414409 PMC4610009

[3] Hoffman AS. Hydrogels for biomedical applications. Advanced Drug Delivery Reviews. 2012;64, Supplement:18–23. 10.1016/j.addr.2012.09.010 11755703

[4] Shin SR, Bae H, Cha JM, Mun JY, Chen Y-C, Tekin H, et al Carbon Nanotube Reinforced Hybrid Microgels as Scaffold Materials for Cell Encapsulation. ACS Nano. 2012;6(1):362–72. 10.1021/nn203711s 22117858 PMC3401631

[5] Liu X, Ma PX. Polymeric Scaffolds for Bone Tissue Engineering. Annals of Biomedical Engineering. 2004;32(3):477–86. 10.1023/B:ABME.0000017544.36001.8e 15095822

[6] Lee KY, Mooney DJ. Hydrogels for Tissue Engineering. Chemical Reviews. 2001;101(7):1869–80. 10.1021/cr000108x 11710233

[7] Karim AA, Bhat R. Fish gelatin: properties, challenges, and prospects as an alternative to mammalian gelatins. Food Hydrocolloids. 2009;23(3):563–76. 10.1016/j.foodhyd.2008.07.002

[8] Elgadir MA, Mirghani MES, Adam A. Fish gelatin and its applications in selected pharmaceutical aspects as alternative source to pork gelatin. JOURNAL OF FOOD AGRICULTURE AND ENVIRONMENT. 2013;11(1):73–9.

[9] Nichol JW, Koshy ST, Bae H, Hwang CM, Yamanlar S, Khademhosseini A. Cell-laden microengineered gelatin methacrylate hydrogels. Biomaterials. 2010;31(21):5536–44. 10.1016/j.biomaterials.2010.03.064 20417964 PMC2878615

[10] Rose J, Pacelli S, Haj A, Dua H, Hopkinson A, White L, et al Gelatin-Based Materials in Ocular Tissue Engineering. Materials. 2014;7(4):3106 10.3390/ma7043106 28788609 PMC5453355

[11] Somboon N, Karrila T, Kaewmanee T, Karrila S. Properties of gels from mixed agar and fish gelatin. International Food Research Journal. 2014;21(2).

[12] Aubin H, Nichol JW, Hutson CB, Bae H, Sieminski AL, Cropek DM, et al Directed 3D cell alignment and elongation in microengineered hydrogels. Biomaterials. 2010;31(27):6941–51. 10.1016/j.biomaterials.2010.05.056 20638973 PMC2908986

[13] Gauvin R, Chen Y-C, Lee JW, Soman P, Zorlutuna P, Nichol JW, et al Microfabrication of complex porous tissue engineering scaffolds using 3D projection stereolithography. Biomaterials. 2012;33(15):3824–34. 10.1016/j.biomaterials.2012.01.048 PMC3766354. 22365811 PMC3766354

[14] Riccardo L, Jetze V, Josep AP, Elisabeth E, Jos M, Miguel AM-T. Biofabrication of tissue constructs by 3D bioprinting of cell-laden microcarriers. Biofabrication. 2014;6(3):035020 10.1088/1758-5082/6/3/035020 25048797

[15] Bertassoni LE, Cecconi M, Manoharan V, Nikkhah M, Hjortnaes J, Cristino AL, et al Hydrogel bioprinted microchannel networks for vascularization of tissue engineering constructs. Lab on a Chip. 2014;14(13):2202–11. 10.1039/C4LC00030G 24860845 PMC4201051

[16] Selimovic S, Oh J, Bae H, Dokmeci M, Khademhosseini A. Microscale Strategies for Generating Cell-Encapsulating Hydrogels. Polymers (Basel). 2012;4(3):1554 Epub 2013/04/30. 10.3390/polym4031554 ; PubMed Central PMCID: PMCPmc3634591. 23626908 PMC3634591

[17] Annabi N, Tsang K, Mithieux SM, Nikkhah M, Ameri A, Khademhosseini A, et al Highly Elastic Micropatterned Hydrogel for Engineering Functional Cardiac Tissue. Adv Funct Mater. 2013;23(39). Epub 2013/12/10. 10.1002/adfm.201300570 ; PubMed Central PMCID: PMCPmc3850066. 24319406 PMC3850066

[18] Eng G, Lee BW, Parsa H, Chin CD, Schneider J, Linkov G, et al Assembly of complex cell microenvironments using geometrically docked hydrogel shapes. Proc Natl Acad Sci U S A. 2013;110(12):4551–6. Epub 2013/03/15. 10.1073/pnas.1300569110 ; PubMed Central PMCID: PMCPmc3607001. 23487790 PMC3607001

[19] Coutinho DF, Sant S, Shakiba M, Wang B, Gomes ME, Neves NM, et al Microfabricated photocrosslinkable polyelectrolyte-complex of chitosan and methacrylated gellan gum. J Mater Chem. 2012;22(33):17262–71. Epub 2013/01/08. 10.1039/c2jm31374j ; PubMed Central PMCID: PMCPmc3534970. 23293429 PMC3534970

[20] Zhao X, Lang Q, Yildirimer L, Lin ZY, Cui W, Annabi N, et al Photocrosslinkable Gelatin Hydrogel for Epidermal Tissue Engineering. Adv Healthc Mater. 2016;5(1):108–18. Epub 2015/04/17. 10.1002/adhm.201500005 ; PubMed Central PMCID: PMCPmc4608855. 25880725 PMC4608855

[21] Shin SR, Aghaei-Ghareh-Bolagh B, Dang TT, Topkaya SN, Gao X, Yang SY, et al Cell-laden microengineered and mechanically tunable hybrid hydrogels of gelatin and graphene oxide. Adv Mater. 2013;25(44):6385–91. Epub 2013/09/03. 10.1002/adma.201301082 ; PubMed Central PMCID: PMCPmc3898458. 23996513 PMC3898458

[22] Heo DN, Ko W-K, Bae MS, Lee JB, Lee D-W, Byun W, et al Enhanced bone regeneration with a gold nanoparticle-hydrogel complex. Journal of Materials Chemistry B. 2014;2(11):1584–93. 10.1039/C3TB21246G 32261377

[23] Gudmundsson M. Rheological Properties of Fish Gelatins. Journal of Food Science. 2002;67(6):2172–6. 10.1111/j.1365-2621.2002.tb09522.x

[24] Padrão J, Silva JP, Rodrigues LR, Dourado F, Lanceros-Méndez S, Sencadas V. Modifying Fish Gelatin Electrospun Membranes for Biomedical Applications: Cross-Linking and Swelling Behavior. Soft Materials. 2014;12(3):247–52. 10.1080/1539445X.2013.873466

[25] Kittiphattanabawon P, Benjakul S, Sinthusamran S, Kishimura H. Gelatin from clown featherback skin: Extraction conditions. LWT—Food Science and Technology. 2016;66:186–92. 10.1016/j.lwt.2015.10.029

[26] Gómez-Guillén MC, Pérez-Mateos M, Gómez-Estaca J, López-Caballero E, Giménez B, Montero P. Fish gelatin: a renewable material for developing active biodegradable films. Trends in Food Science & Technology. 2009;20(1):3–16. 10.1016/j.tifs.2008.10.002

[27] Irwandi J, Faridayanti S, Mohamed ESM, Hamzah MS, Torla HH, Che Man YB. Extraction and characterization of gelatin from different marine fish species in Malaysia. International Food Research Journal. 2009;16(3):381–90.

[28] Benton JA, DeForest CA, Vivekanandan V, Anseth KS. Photocrosslinking of gelatin macromers to synthesize porous hydrogels that promote valvular interstitial cell function. Tissue Eng Part A. 2009;15(11):3221–30. Epub 2009/04/21. 10.1089/ten.TEA.2008.0545 ; PubMed Central PMCID: PMCPmc2783792. 19374488 PMC2783792

[29] Habeeb AF. Determination of free amino groups in proteins by trinitrobenzenesulfonic acid. Anal Biochem. 1966;14(3):328–36. Epub 1966/03/01. 10.1016/0003-2697(66)90275-2 . 4161471

[30] Du YA, Lo E, Ali S, Khademhosseini A. Directed assembly of cell-laden microgels for fabrication of 3D tissue constructs. Proceedings of the National Academy of Sciences of the United States of America. 2008;105(28):9522–7. 10.1073/pnas.0801866105 . 18599452 PMC2474514

[31] Engler AJ, Sweeney HL, Discher DE, Schwarzbauer JE. Extracellular matrix elasticity directs stem cell differentiation. J Musculoskelet Neuronal Interact. 2007;7(4):335 . 18094500

[32] Gudmundsson M, Hafsteinsson H. Gelatin from cod skins as affected by chemical treatments. Journal of Food Science. 1997;62(1):37–9. 10.1111/j.1365-2621.1997.tb04363.x

[33] Eastoe JE. The amino acid composition of fish collagen and gelatin. Biochemical Journal. 1957;65(2):363–8. PMC1199877. 10.1042/bj0650363 13403916 PMC1199877

[34] Farris S, Schaich KM, Liu L, Piergiovanni L, Yam KL. Development of polyion-complex hydrogels as an alternative approach for the production of bio-based polymers for food packaging applications: a review. Trends in Food Science & Technology. 2009;20(8):316–32. 10.1016/j.tifs.2009.04.003

[35] Muyonga JH, Cole CG, Duodu KG. Extraction and physico-chemical characterisation of Nile perch (Lates niloticus) skin and bone gelatin. Food hydrocolloids. 2004;18(4):581–92. 10.1016/j.foodhyd.2003.08.009

[36] Araghi M, Moslehi Z, Mohammadi Nafchi A, Mostahsan A, Salamat N, Daraei Garmakhany A. Cold water fish gelatin modification by a natural phenolic cross-linker (ferulic acid and caffeic acid). Food Sci Nutr. 2015;3(5):370–5. Epub 2015/09/26. 10.1002/fsn3.230 ; PubMed Central PMCID: PMCPmc4576961. 26405523 PMC4576961

[37] Badii F, Howell NK. Fish gelatin: Structure, gelling properties and interaction with egg albumen proteins. Food Hydrocolloids. 2006;20(5):630–40. 10.1016/j.foodhyd.2005.06.006

[38] Okay O. General Properties of Hydrogels In: Gerlach G, Arndt K-F, editors. Hydrogel Sensors and Actuators: Engineering and Technology. Berlin, Heidelberg: Springer Berlin Heidelberg; 2010 p. 1–14.

[39] Kim SW, Bae YH, Okano T. Hydrogels: swelling, drug loading, and release. Pharm Res. 1992;9(3):283–90. Epub 1992/03/01. . 1614957 10.1023/a:1015887213431

[40] Omidian H, Hasherni S-A, Askari F, Nafisi S. Swelling and crosslink density measurements for hydrogels. Iranian J of Polymer Science and Technology Vol. 1994;3(2).

[41] Ganji F, Vasheghani-Farahani S, Vasheghani-Farahani E. Theoretical description of hydrogel swelling: a review. Iran Polym J. 2010;19(5):375–98.

[42] Selimović Š, Oh J, Bae H, Dokmeci M, Khademhosseini A. Microscale strategies for generating cell-encapsulating hydrogels. Polymers. 2012;4(3):1554–79. 10.3390/polym4031554 23626908 PMC3634591

[43] Ahearne M. Introduction to cell–hydrogel mechanosensing. Interface focus. 2014;4(2):20130038 10.1098/rsfs.2013.0038 24748951 PMC3982445

[44] Hutson CB, Nichol JW, Aubin H, Bae H, Yamanlar S, Al-Haque S, et al Synthesis and characterization of tunable poly (ethylene glycol): gelatin methacrylate composite hydrogels. Tissue Engineering Part A. 2011;17(13–14):1713–23. 10.1089/ten.TEA.2010.0666 21306293 PMC3118706

[45] Leor J, Amsalem Y, Cohen S. Cells, scaffolds, and molecules for myocardial tissue engineering. Pharmacology & therapeutics. 2005;105(2):151–63. 10.1016/j.pharmthera.2004.10.003 15670624

[46] Khademhosseini A, Vacanti JP, Langer R. Progress in tissue engineering. Scientific American. 2009;300(5):64–71. 10.1038/scientificamerican0509-64 19438051

[47] Schuurman W, Levett PA, Pot MW, van Weeren PR, Dhert WJ, Hutmacher DW, et al Gelatin‐methacrylamide hydrogels as potential biomaterials for fabrication of tissue‐engineered cartilage constructs. Macromolecular bioscience. 2013;13(5):551–61. 10.1002/mabi.201200471 23420700

[48] Wells RG. The role of matrix stiffness in regulating cell behavior. Hepatology. 2008;47(4):1394–400. 10.1002/hep.22193 18307210

[49] Solon J, Levental I, Sengupta K, Georges PC, Janmey PA. Fibroblast adaptation and stiffness matching to soft elastic substrates. Biophysical journal. 2007;93(12):4453–61. 10.1529/biophysj.106.101386 18045965 PMC2098710

[50] Zhu J, Marchant RE. Design properties of hydrogel tissue-engineering scaffolds. Expert review of medical devices. 2011;8(5):607–26. 10.1586/erd.11.27 PMC3206299. 22026626 PMC3206299

[51] Klotz BJ, Gawlitta D, Rosenberg AJ, Malda J, Melchels FP. Gelatin-Methacryloyl Hydrogels: Towards Biofabrication-Based Tissue Repair. Trends Biotechnol. 2016 Epub 2016/02/13. 10.1016/j.tibtech.2016.01.002 . 26867787 PMC5937681

